# The procalcitonin/albumin ratio as an early diagnostic predictor in discriminating urosepsis from patients with febrile urinary tract infection

**DOI:** 10.1097/MD.0000000000011078

**Published:** 2018-07-13

**Authors:** Xin Luo, Xiang Yang, Jiexian Li, Ge Zou, Yufeng Lin, Guoqiang Qing, Ruilin Yang, Weixiang Yao, Xuying Ye

**Affiliations:** a Department of Urology; b Department of Gynecology, Panyu Central Hospital, Guangzhou, China.

**Keywords:** albumin, diagnostic, febrile urinary tractinfection, procalcitonin, urosepsis

## Abstract

Discrimination between urosepsis and febrile urinary tract infections is important in therapeutic decision-making to indicate suitable treatments to avoid sepsis-related organ failure. Accurate diagnosis is time-consuming and susceptible to false-positive results. Moreover, patient responses to urosepsis are complex and varied. Therefore, this study aimed to develop a new, early diagnostic predictor that could discriminate between patients with urosepsis and those with febrile urinary tract infections using a combination of initial procalcitonin and albumin levels.

We conducted a retrospective study involving 140 patients with febrile urinary tract infections from January 2013 to December 2017. Univariate and multivariate logistic analyses were performed to identify the independent risk factors for differentiating urosepsis from febrile urinary tract infection. A receiver operating characteristic (ROC) curve analysis was conducted to compare the predictive accuracy of the procalcitonin/albumin ratio.

Patients in the urosepsis group had higher procalcitonin/albumin ratios compared to those in the febrile urinary tract infection group [2.254 (0.978, 6.299) vs 0.021 (0.004, 0.095); *P < *.001]. Based on multivariate logistic analysis, the procalcitonin/albumin ratio [adjusted odds ratio (OR) 1.029, 95% confidence interval (CI) 1.013–1.045, *P < *.001] was an independent predictor of urosepsis, which allowed for differentiation from patients with febrile urinary tract infections. The area under the ROC curve (AUC) for the procalcitonin/albumin ratio was 0.937 (95% CI, 0.894–0.980); *P < *.001. The sensitivity and specificity of the procalcitonin/albumin ratio cut-off values (>0.44) were 84.62% and 96.00%, respectively. Moreover, in the subset of 65 patients with urosepsis, the procalcitonin/albumin ratio in the uroseptic shock group was higher than in the group of patients without uroseptic shock [5.46 (1.43, 6.58) vs 1.24 (0.63, 4.38); *P = *.009].

Our study demonstrates that the procalcitonin/albumin ratio is an early diagnostic predictor that can discriminate between urosepsis and febrile urinary tract infection. Additionally, in patients with urosepsis, those with higher procalcitonin/albumin ratios were more prone to uroseptic shock. Our findings suggest that the procalcitonin/albumin ratio is a rapid and relatively low-cost biomarker that can be used in clinical practice.

## 1 Introduction

Urinary tract infection (UTI) is one of the most common diseases encountered by urologists. Febrile UTI (fUTI) typically represents acute pyelonephritis or acute prostatitis, but it may also represent urosepsis.^[1,2]^ Urosepsis is life-threatening organ dysfunctions caused by dysregulated host responses to infections originating in the urinary tract and/or male genital organs.^[3,4]^ The overall mortality rate for urosepsis can range from 7.5% to 30%.^[5,6]^ Moreover this condition can prolong the length of hospital stays, increase costs and worsen a patient's overall prognosis, however, these adverse consequences can be alleviated by early diagnosis and treatment.^[7]^ The ability to immediately discriminate urosepsis from fUTI is important in therapeutic decision-making and provides these patients with suitable treatments that can avoid sepsis-related organ failure. However, the response to urosepsis is complex and not all patients with fUTI display similar signs or symptoms. The diagnosis of urosepsis is time-consuming and can only be confirmed with blood and urine culture results.^[8]^ The process of diagnosing these infections can take anywhere from 24 to 72 hours, based on the time required to obtain the culture results. Additionally, there may be false positive results because of contamination. Therefore, rapid and efficient diagnostic methods for discriminating urosepsis from fUTI are necessary. Procalcitonin (PCT) is a marker of systemic inflammation and thus, may help to predict bacteraemia,^[9,10]^ which is regarded as a diagnostic marker of sepsis in critically ill patients.^[11]^ Currently, PCT levels are widely used and have been validated as indicators of urosepsis. The test for PCT exhibits good sensitivity,^[12,13]^ but sometimes exhibits poor specificity. Several reports have observed that PCT levels can be increased in noninfectious conditions and may remain low during actual infections. ^[14]^ Albumin is also a potent marker of outcomes in infection-related diseases, as its levels tend to decrease during acute phase infections.^[15,16]^

The correlation between the ratio of PCT to albumin in the early diagnosis of urosepsis in patients with fUTI is unclear, but there was no study that had report this correlation. We speculated that the PCT/albumin ratio could be used as a predictive marker for the early diagnosis of urosepsis in patients with fUTI. The aim of this study was to investigate the use of the PCT/albumin ratio as an early diagnostic predictor for discriminating between urosepsis and fUTI. We compared the PCT/albumin ratio to currently available biomarkers such as blood leucocyte counts and C-reactive protein (CRP) levels.

## 2 Methods

### 2.1 Ethical approval

This study was a retrospective review of patients treated at our hospital. It was approved by the ethics committees of Panyu Central Hospital, Guangzhou, China. (Number: K20170001).

### 2.2 Patient selection

We conducted a retrospective study of patients with febrile urinary tract infections admitted to Guangzhou Panyu Central Hospital, China, a 1300-bed general hospital, from January 2013 to December 2017. The inclusion criteria were: fever (≥38.0°C), age ≥18 years, a history of fever or shaking chills within 24 hours of presentation, at least 1 symptom of UTI (flank pain or dysuria), and leucocyturia, or a positive urine culture. The exclusion criteria were: patients aged < 18 years, pregnancy, history of kidney transplantation, hemodialysis or peritoneal dialysis, patients with missing data, and patients with a disease that could affect the PCT level (such as thyroid disease). Urosepsis was defined as sepsis caused by an infection of the urinary tract and/or male genital organs ^[5]^ According to our standard definition, the patients with fUTI were considered to have urosepsis if they had a bacterial infection proven by blood cultures or diagnosed clinically, and exhibited at least 2 of the following 4 criteria: fever (>38°C) or hypothermia (<36°C); tachycardia (>90 bpm); tachypnea (>20 respirations/minute); or leucopoenia (leucocyte count <4.0 × 10^9^/L), leucocytosis (leucocyte count >12.0 × 10^9^/L) or a left-ward shift (>10% immature granulocytes). Patients were categorized into 2 groups according to the presence or absence of urosepsis. Blood samples for measuring CRP levels, leucocytes, platelets, albumin, and PCT were taken on admission, Demographics, diagnoses, and clinical and laboratory findings were recorded. All patients’ medical records were reviewed, and the relevant clinical and biological data were collected. Basic characteristics, including demographic information and pre-existing comorbidities including diabetes, chronic pulmonary disease, and renal failure were collected.

### 2.3 Laboratory protocol

Complete blood count (CBC), serum albumin, CRP, and PCT levels were checked upon admission to the hospital. PCT levels were measured using the automatic analyser VIDAS BRAHMS PCT package insert (BIOMÉ RIEUX), according to the manufacturer's instructions. The lower limit of detection of the assay was 0.05 ng/mL and the functional assay sensitivity was 0.09 ng/mL. CBC, serum albumin, and CRP were monitored using an automatic nephelometer (Beckman Coulter, Pasadena, CA). Normal ranges were 0 to 6 mg/L for CRP and 35.0 to 53.0 g/L for albumin.

### 2.4 Statistical analysis

We conducted a statistical analysis that compared the general information of each group using the independent samples *t*-test and Mann–Whitney *U* test for continuous variables, and the χ^2^ test for categorical variables. Categorical variables are expressed as numbers and percentages for the description of the basic characteristics and the continuous variables are expressed as means ± standard deviation or medians (upper and lower quartile). Sensitivity and specificity, as well as positive and negative predictive values were calculated. The statistical analyses were performed using SPSS version 17.0 (SPSS, Chicago, IL). Unconditional logistic regression analysis was used to determine whether each variable was an independent factor in urosepsis. Covariates for the multivariate logistic regression analysis were selected based on a *P*-value <.05 in a univariate analysis. Variables were considered significant if *P < *.05, and the results are presented as odds ratio with 95% confidence intervals (CIs). Prediction accuracy was assessed using the area under the receiver operating characteristic (ROC) curve. Cut-off values showing the greatest accuracy were determined using sensitivity/specificity.

## 3 Results

A total of 140 patients with febrile urinary tract infections were enrolled in this study. Sixty-five patients with urosepsis were identified as the urosepsis group, and 75 patients without urosepsis were identified as the fUTI group. The mean age of the enrolled patients was 58.47 ± 16.99 years, and 46 (32.86%) were men. Diabetes mellitus was present in 13.57% of the patients and 19.29% of the patients had renal failure (Table 1). Fever or shaking chills were present in 72.86% of the patients, 84.29% of the patients had flank pain, odynuria or dysuria, and 21.43% of the patients had hypotension. The positive urine and blood cultures were present in 50 (35.71%) and 34 (21.43%) patients, respectively (Table 2). All patients’ blood samples were collected within 24 hours of admission to the hospital. The CRP levels, leucocyte counts, and PCTs were significantly higher in patients with urosepsis than in those with fUTI. The mean CRP was 164.65 ± 66.79 (7.81–311.8) mg/L and 104.48 ± 75.24 (0.6–200) mg/L, respectively. The mean concentration of leucocytes was 15.21 (11.01, 23.5) and 10.22 (6.93, 13.36), respectively. The mean concentration of PCT was 63.84 (30.28, 200) and 0.72 (0.15, 3.35), respectively. The albumin and platelets on admission were significantly lower in patients with urosepsis than in those with fUTI. The mean concentration of albumin was 29.74 ± 4.69 (17–40.9) g/L and 35.12 ± 4.23 (26.8–42.8) g/L, respectively. The mean platelet counts were 120 (75, 203) and 206 (164.75, 266.25), respectively. Among the biomarkers, the PCT/albumin ratio was significantly higher in patients with urosepsis compared to patients with fUTI [2.254 (0.978, 6.299) vs 0.021 (0.004, 0.095); *P < *.01] (Table 1).

**Table 1:**
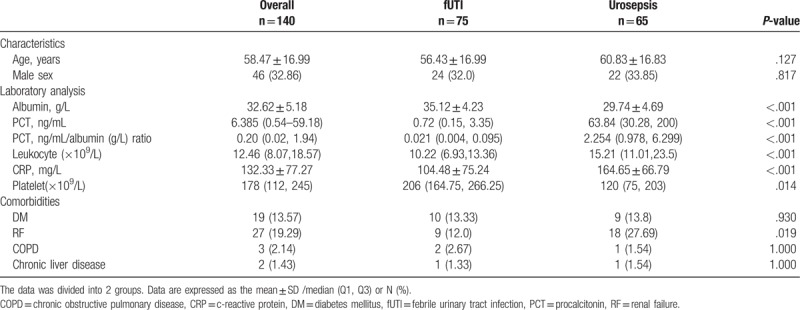
Baseline features of the enrolled patients.

**Table 2:**
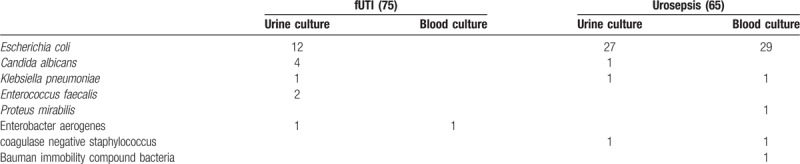
The causative organisms in the collected blood and urine cultures of the 2 groups.

### 3.1 The PCT/albumin ratio as an independent predictor of urosepsis in patients with fUTI

In an unconditional logistic regression analysis, renal failure, CRP, PCT, albumin, leucocytes, and the PCT/albumin ratio at admission were significantly associated with urosepsis. Furthermore, in a multivariate logistic regression analysis, the PCT/albumin ratio (*P < *.001) was the only significant factor indicative of urosepsis. The OR of the PCT/albumin ratio was 1.029 (95% CI, 1.013–1.045) (Table 3).

**Table 3:**
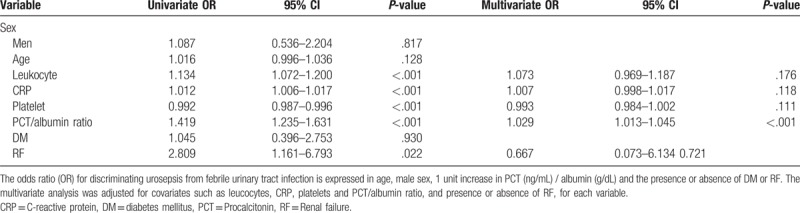
Univariate and multivariate logistic analyses for discriminating urosepsis from febrile urinary tract infection.

### 3.2 Diagnostic performance of the PCT/albumin ratio

**Figure 1:**
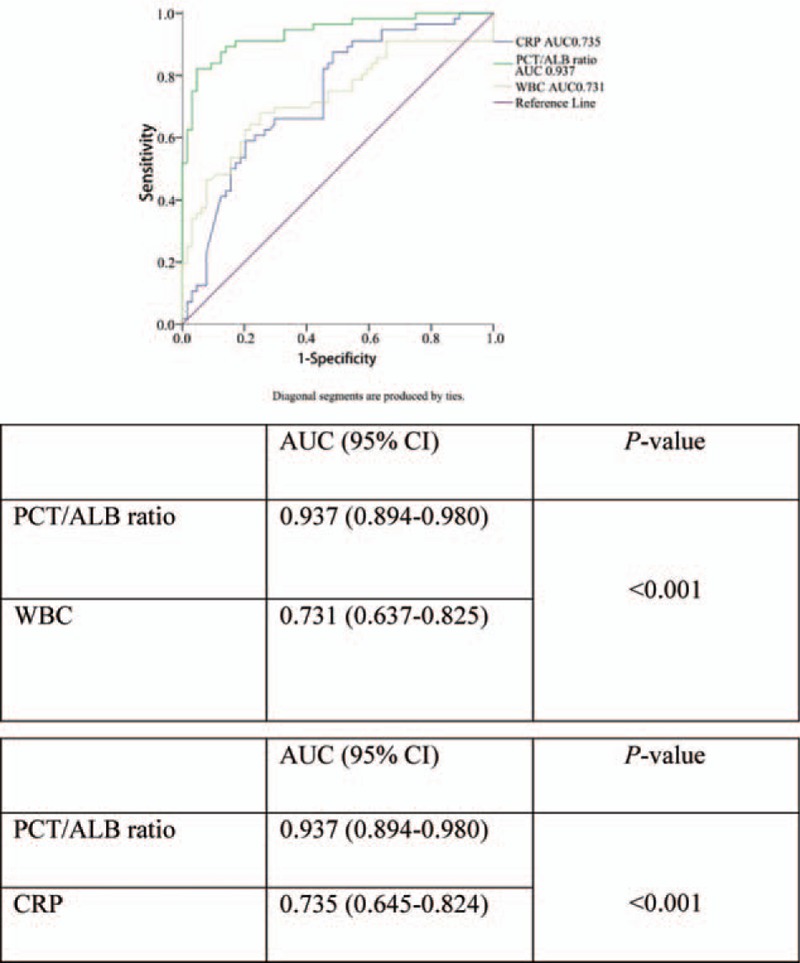
Receiver operating characteristic (ROC) curve of the PCT/Albumin ratio, WBC and CRP for discriminating urosepsis from febrile urinary tract infection. ALB = albumin, CRP = C-reactive protein, PCT = procalcitonin, ROC=receiver operating characteristic.

The predictability of discriminating urosepsis from fUTI was assessed using the area under the ROC curve (AUC) (Fig. 1). The AUC for the PCT/albumin ratio at admission (0.937 [95% CI, 0.894–0.980; *P < *.001]) was significantly greater than for CRP (0.735 [95% CI, 0.645–0.824; *P < *.001]) or the leucocyte count (0.731 [95% CI, 0.637–0.825; *P < *.001]). Moreover, a mountain curve analysis determined that the most sensitive and specific cut-off for the PCT/albumin ratio was 0.44 (Table 4). In addition, the sensitivity and specificity of the PCT/albumin ratio cut-off values (>0.44) were 84.62% and 96.00%, respectively, while the CRP and leucocyte cut-off values were less specific (sensitivity and specificity of 87.50% and 50.77%, and sensitivity and specificity of 64.62% and 74.32%), respectively (Table 4). The analysis demonstrated that the PCT/albumin ratio as an early diagnostic predictor in discriminating urosepsis from fUTI with an elevated PCT/albumin ratio of 0.44 (Table 3).

**Table 4:**
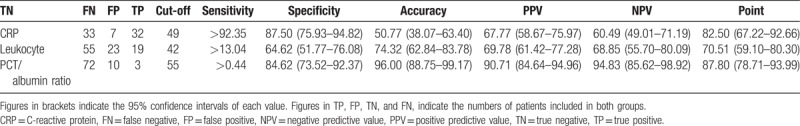
Comparison of the diagnostic performance of each predictor for discriminating urosepsis from febrile urinary tract infection.

### 3.3 Additional analyses

Additional analyses were conducted to determine whether the PCT/albumin ratio was associated with the severity of infection in patients with urosepsis. Therefore, a separate analysis was performed involving only patients with urosepsis (n = 65). We divided those patients into 2 groups according to the presence or absence of uroseptic shock. In the uroseptic shock group, the CRP and PCT levels were significantly higher, while the albumin levels and platelet counts were significantly lower. The mean concentrations of albumin were 28.07 ± 4.96 g/L in the uroseptic shock group and 31.26 ± 3.88 g/L in the group without uroseptic shock. The PCT/albumin ratio was significantly higher in the uroseptic shock compared to those with urosepsis without shock [5.46 (1.43, 6.58) vs 1.24 (0.63, 4.38); *P = *.003] (Table 5).

**Table 5:**
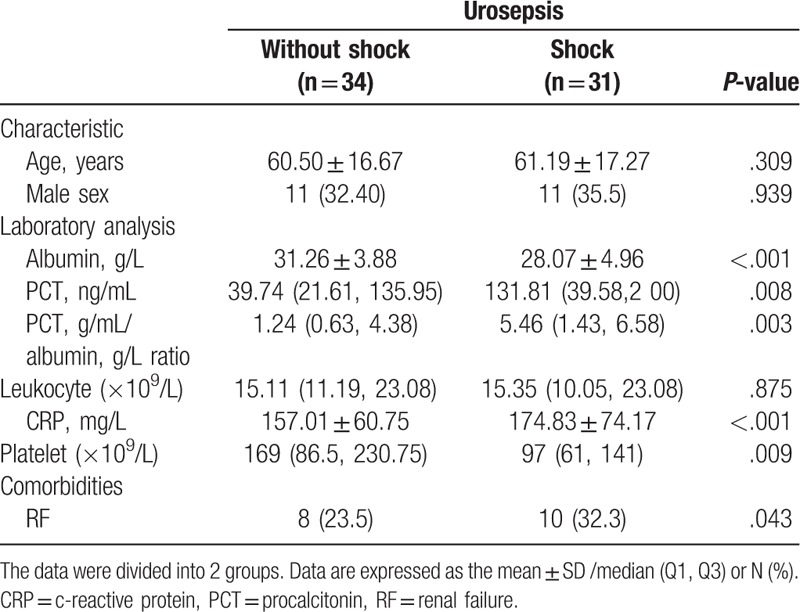
Baseline features of the patients with urosepsis.

## 4 Discussion

The main finding of our study was that the PCT/albumin ratio at the time of admission can be used as an early diagnostic predictor for discriminating urosepsis from fUTI, and is an improvement over the traditional and widespread biomarkers of systemic infection such as CRP and leucocyte count. Our findings demonstrated a significantly higher AUC value than for CRP and leucocytes. To our knowledge, this is the first study assessing the utility of the PCT/albumin ratio for discriminating between urosepsis and fUTI.

CRP and leucocyte are generally regarded as useful markers for the diagnosis of sepsis, and are usually used together with blood cultures. However, such methods are sometimes inadequate for early diagnosis, since it takes anywhere from 24 to 72 h to obtain the culture results. This delay in diagnosis can be detrimental to the overall health status of the patient. Therefore, more rapid and efficient diagnostic methods were necessary. The neutrophil-lymphocyte count ratio (NLCR) has previously been described as a predictor of bacteraemia.^[17–21]^ Lars Ljungström^[11]^ reported that NLCR or PCT alone exhibited equivalent performance in confirming the presence of bacterial sepsis in less severe septic conditions.

PCT accurately predicts the presence of bacteraemia and bacterial load in patients with febrile UTI.^[22]^ Some studies have shown that a PCT >2 ng/mL has >90% specificity for sepsis or the progression to sepsis, whereas <0.5 ng/mL PCT levels are not associated with sepsis.^[23]^ We also found that the CRP, leukocyte count, and PCT upon admission were significantly higher in patients with urosepsis than in those with fUTI. Particularly with the PCT levels, 26.2% of patients (n = 17) with urosepsis exceeded the 200 ng/mL threshold, which was much higher than the values needed to suggest urosepsis.^[24]^ This finding was likely because those patients with urosepsis had fewer basic diseases and were overall younger in age. The PCT levels were higher in our study for the following possible reasons: most of the patients with UTI were infected with gram-negative organisms, which causes higher peak PCT values than infections caused by gram-positive organisms,^[25]^ different observation times may have resulted in different PCT optimal cut-off values to diagnose sepsis,^[26]^ different test methods and reagents may have been used,^[27]^ but these was the same in our hospital, all of the patients’ PCT levels were taken on admission, thereby reducing bias.

In the past, serum albumin levels were considered to be indicators of nutritional status, however, more recently, literature has shown that serum albumin concentration, severity of the disease, and the mortality rate have a close relationship. Hypoalbuminaemia can be seen as an independent risk factor for morbidity and mortality of illness.^[28]^ Albumin levels also are indicative of inflammatory status.^[29,30]^ Low albumin concentrations reflect low serum protein levels, leading to low bactericidal activity of serum and predisposing to bacterial infections.^[31]^ Hypoalbuminaemia may be related to inflammatory changes in the sepsis process or be a reflection of poor baseline nutrition levels. Although the precise mechanisms have not been fully described, serum albumin has protective effects such as antioxidant activity, maintenance of physiologic homeostasis, and anti-inflammatory effects.^[32,33]^ Therefore, these protective biologic functions may be impaired in conditions of hypoalbuminaemia, and therefore, increased morbidity and mortality can consequently develop in severely septic patients.^[34]^ This theory is consistent with our study findings since the levels of albumin on admission were significantly lower in patients with urosepsis than in those with fUTI. Moreover, as the infection became more severe the albumin levels decreased significantly. This is consistent with the report by Falguera et al^[35]^ who considered that low serum albumin is a predictor in patients with an infection such as community acquired pneumonia. Thus, to improve the potential predictive capabilities of each single factor, PCT and albumin were combined. The combination of these markers enabled inflammatory and nutritional factors to be merged, which may have enhanced its predictive value. We consider the PCT/albumin ratio, in theory, to be a superior predictive factor compared to other indicators of inflammation or nutrition. In our study, the PCT/albumin ratio upon admission was significantly increased in patients with urosepsis patients compared to those patients with fUTI. Moreover, the level was much higher in patients with uroseptic shock than in patients with urosepsis without shock. Therefore, we speculated that the PCT/albumin ratio was associated with the severity of infection.

In a univariate analysis, CRP, leucocyte count and platelet count at admission were significantly associated with urosepsis. Furthermore, in a multivariate analysis, only the PCT/albumin ratio at admission was independent predictors of urosepsis, which the ROC curve demonstrated a significantly higher than CRP and Leucocyte. Recently, little study has evaluated the diagnosis significance of the PCT/albumin ratio in discriminating urosepsis from Patients with fUTI. Moreover, we estimated the optimal cut-off value of PCT/albumin ratio to distinguish the patients at-risk for urosepsis from those with fUTI, thus maximizing sensitivity without a substantial loss of specificity. The diagnostic accuracy of the statistical models generated was further validated by the generation of a ROC curve. This curve revealed that the optimal cut-off value for the PCT/albumin ratio for discriminating urosepsis from fUTI was >0.44, with 84.62% sensitivity and 96.00% specificity (Table 3). The PCT/albumin ratio >0.44 had the best sensitivity and specificity in predicting urosepsis. Furthermore, the PCT/albumin ratio may be an indicator of the severity of urosepsis. In our study, we found that the PCT/albumin ratio was significantly higher in uroseptic shock compared to urosepsis without shock [5.46 (1.43, 6.58) vs 1.24 (0.63, 4.38); *P = *.003]. PCT levels are considered to be related to infection, but sometimes it is increased in non-infectious conditions and may remain low during infections. As we know, albumin levels tend to decrease in patients with infection. Therefore, we combined the 2 biomarkers to improve the sensitivity and specificity of early the diagnosis of urosepsis before the onset of hypotension or thrombocytopenia. Our results show that the PCT/albumin ratio is a useful predictor because it allows for the prompt diagnosis of urosepsis in patients with fUTI without needing to wait for blood culture results.

This study also had several limitations. First, it was a retrospective study and therefore, bias was likely. Second, the data were collected from a single centre. As such, it is possible that the results could differ from those of other centres, and the predictive probability could have been overestimated compared to a prospective study. Additional prospective studies with larger populations involving multiple centres are necessary to validate our conclusion.

## 5 Conclusion

A procalcitonin/albumin ratio exceeding 0.44 is valuable for discriminating urosepsis from fUTI, and it may be superior to traditional biomarkers such as CRP levels and leucocyte counts. Our findings suggest that the procalcitonin/albumin ratio is a rapid biomarker and is low-cost in clinical practice. These findings could be confirmed by prospective studies with larger populations involving multiple centres.

## Acknowledgments

We thank Yuantin Liu (Statistics Office of Panyu Central Hospital, Guangzhou, China) for statistical analysis.

## Author contributions

**Conceptualization:** xin luo.

**Data curation:** Yufeng Lin.

**Formal analysis:** xin luo, Ge Zou, Weixiang Yao.

**Investigation:** xin luo, Xuying Ye.

**Methodology:** Yufeng Lin.

**Project administration:** Guoqiang Qing.

**Resources:** Ge Zou, Guoqiang Qing.

**Supervision:** xin luo, Jiexian Li.

**Validation:** Jiexian Li, Ruilin Yang.

**Visualization:** Ruilin Yang.

**Writing – original draft:** xin luo.

**Writing – review & editing:** Xiang Yang.
